# Removal of an aspirated dental bridge in the right main bronchus after endotracheal tube extubation: A case report

**DOI:** 10.1097/MD.0000000000042225

**Published:** 2025-05-30

**Authors:** Yeonjeong Heo, Oh Beom Kwon, Da Hye Moon, Seon-Sook Han, Woo Jin Kim, Seung-Joon Lee, Jeongwon Heo

**Affiliations:** a Department of Internal Medicine, Kangwon National University, Chuncheon, Korea.

**Keywords:** aspiration, case report, dental bridge, foreign body in bronchus

## Abstract

**Rationale:**

Foreign body aspiration into bronchial tubes is a serious medical problem. Here, we report a case of bronchial foreign body removal using flexible bronchoscopy with a balloon catheter and cryoadhesions.

**Patient concerns:**

The patient was diagnosed with septic shock and was treated in the intensive care unit. After improvement and extubation, chest radiography revealed a bronchial foreign body, and atelectasis in the right lower lobe was confirmed.

**Diagnoses:**

Chest radiography revealed the presence of a radiopaque foreign body in the right main bronchus.

**Interventions:**

A balloon catheter was used to dislodge the foreign body with a retrograde pull, and the foreign body was removed using cryoadhesions.

**Outcomes:**

The foreign body was successfully removed using a balloon catheter and cryoadhesions, without major complications. After surgical removal of the bronchial foreign body, chest radiography revealed resolution of right lower lobe atelectasis, and no dyspnea or sputum production was observed. Subsequently, the patient’s condition improved, and he was discharged from the hospital.

**Lessons:**

An increase in dental treatments has led to the development of various artificial dental structures. Therefore, rechecking these artificial structures upon intensive care unit admission and extubation may help prevent their aspiration.

## 1. Introduction

Aspiration of foreign bodies into the bronchial tree frequently occurs in both adults and children and can cause serious medical problems.

Rigid bronchoscopy is primarily used to remove foreign bodies from the bronchus. This is because it has a larger working channel than flexible bronchoscopy, allowing for better ventilation and effective use of additional instruments. However, flexible bronchoscopy does not require general anesthesia and can be completed within a relatively short period. Additionally, foreign bodies found in the bronchus of adults have been reported to be successfully removed using flexible bronchoscopy.^[1]^

Traditionally, bronchial foreign body removal is performed using rigid bronchoscopy.^[2]^ However, in this case, the difference lies in the use of a flexible bronchoscope to remove a bronchial foreign body in an adult. In this case, the bronchial foreign body that was tightly lodged in the bronchial tree was first pulled up to the right main bronchus using a balloon catheter and subsequently removed using cryoadhesions.

## 2. Case report

**Figure 1.**
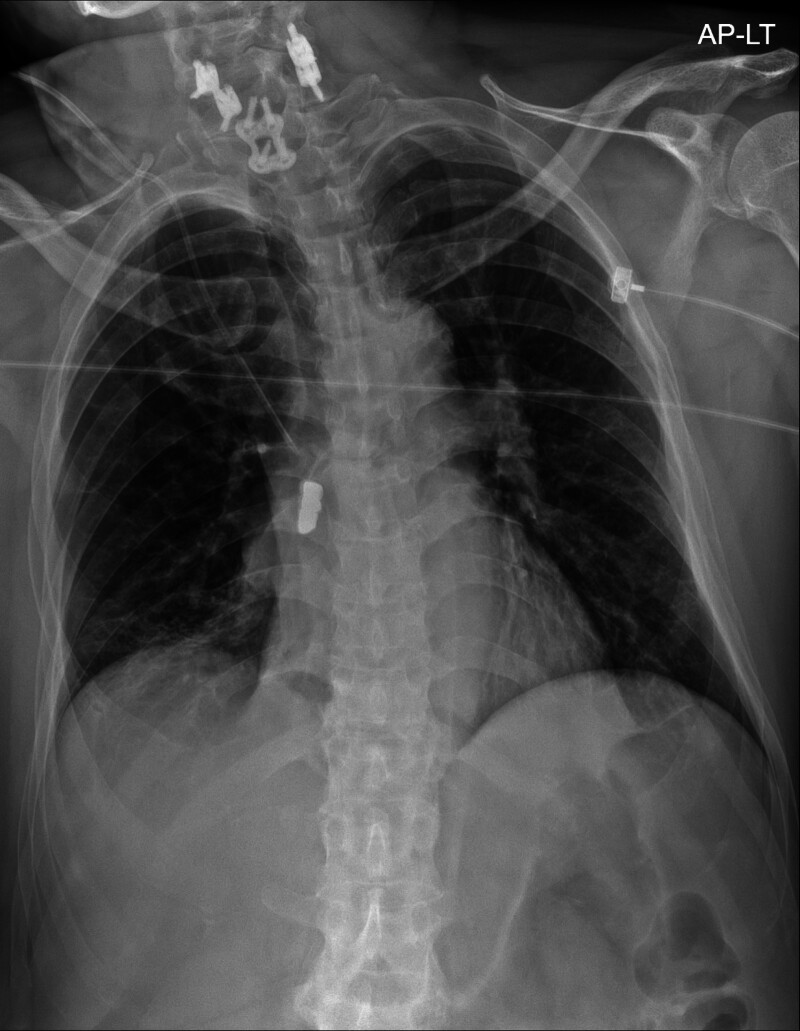
Chest radiography reveals a radiopaque foreign body in the right main bronchus region.

A 62-year-old male patient was admitted to the intensive care unit (ICU) owing to septic shock caused by a urinary tract infection (UTI). His medical history included tetraplegia resulting from a cervical spinal cord injury (C6-7). He had a history of bladder stones and had been hospitalized multiple times for recurrent UTIs. While treating the UTI with antibiotics, the patient underwent cystolitholapaxy under general anesthesia for bladder stones. Shortly after surgery and extubation, the patient was observed to have persistently decreased consciousness and reduced oxygen saturation, leading to reintubation and transfer to the ICU. Following the surgical procedure, the patient was transferred to the recovery room. Despite observation, the patient’s consciousness did not recover as expected. After extubation, the patient developed labored breathing with a subsequent decrease in oxygen saturation. Concurrent hypotension was also noted, necessitating reintubation in the recovery area. The sudden postoperative deterioration was attributed to the progression of a preexisting UTI, which had not been adequately controlled and subsequently led to septic shock. After transfer to the ICU, his condition worsened to septic shock, requiring broad-spectrum antibiotics and continued treatment with mechanical ventilation. The patient was extubated 11 days after intubation and mechanical ventilation. During this period, we discovered that a three-tooth bridge located in the patient’s mouth had passed through the vocal cords, traversed the trachea, and lodged in the right main bronchus (Fig. 1).

**Figure 2.**
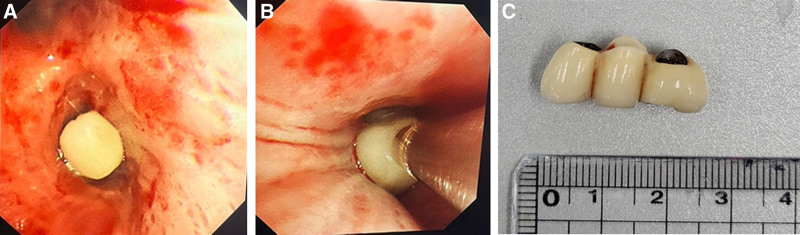
Flexible bronchoscopy images. (A) Foreign body is observed in the right lower lobar bronchus. (B) The bronchial foreign body is removed via cryoadhesion using a cryoprobe. (C) Foreign body: approximately 3 cm three-tooth bridge.

And when a physical examination was performed on the patient, no specific findings such as fixed wheezing were identified. Routine chest radiography revealed a radiopaque foreign body, as shown in Figure 1; however, the patient was asymptomatic. Nevertheless, owing to the large size of the foreign body and the presence of right lower lobe (RLL) atelectasis, we decided to remove it using flexible bronchoscopy based on the pulmonologist’s opinion. The foreign body was removed using a flexible bronchoscope (BF-1TQ290; Olympus, Tokyo, Japan). As the patient was conscious, sedation was performed using midazolam and fentanyl before bronchoscopy was started. During the procedure, oxygen saturation was maintained at 99% to 100% without supplemental O_2_. Bronchoscopy revealed a foreign body in the RLL (Fig. 2). We used a balloon (B5-C2; Olympus, Tokyo, Japan) to dislodge the three-tooth bridge that was tightly lodged in the RLL bronchus and moved it to the area around the carina. Then, we used a flexible cryoprobe with a diameter of 2.4 mm and a length of 900 mm (20416-032, Erbokryo-CA, Erbe, Germany) to remove the bronchial foreign body. The foreign body was too large to be removed using standard forceps; therefore, we decided to use a cryoprobe. The method of using cryoadhesion for foreign body removal proved to be effective; however, because of the cryosensitive nature of the material, considerable time was required to position and rotate it for removal. After the bronchial foreign body removal procedure, chest radiography revealed that the RLL atelectasis had resolved, and there were no symptoms of dyspnea or sputum production. Following the procedure, the patient’s condition stabilized, and he was transferred from the ICU to the general ward. The patient’s condition continued to improve, and he was subsequently discharged.

## 3. Discussion

In this case, after extubation following intubation due to septic shock, a dental bridge that had been in the patient’s mouth dislodged and entered the bronchus, as confirmed using chest radiography. The patient, who had tetraplegia and spent most of his time lying down, likely experienced minimal or no symptoms during foreign body aspiration. Routine chest radiography performed in the ICU revealed a dental bridge in the right main bronchus and RLL atelectasis. After removing the foreign body using bronchoscopy, the atelectasis improved, and the radiopaque foreign body previously seen on the radiograph was no longer visible.

Foreign body aspiration into the trachea occurs frequently in adults aged > 70 years.^[3]^ Common risk factors in this age group include decreased consciousness, alcohol intoxication, sedative drug use, poor dentition, swallowing impairment, Parkinsonism, and general anesthesia.^[4]^ Symptoms of foreign body aspiration into the trachea range from acute asphyxiation (with or without complete airway obstruction) to cough, dyspnea, choking, or fever. In adults, foreign bodies are most commonly found in the right-sided airway.^[5]^

In this case, the patient was positioned with the head elevated by > 30° in the ICU and had an upright upper body when the endotracheal tube was removed, allowing the foreign body to settle in the RLL bronchus. The clinical symptoms of foreign body aspiration can vary depending on the size and location of the foreign body. Complications of airway foreign bodies include atelectasis, pneumonia, respiratory distress, bronchiectasis, cardiopulmonary arrest, and pneumothorax.

Chest computed tomography is often performed when foreign body aspiration is suspected; however, the chest radiograph of our patient was sufficiently clear to identify the bronchial foreign body; therefore, additional chest computed tomography was not performed. Furthermore, the aspirated dental bridge was radiopaque, making it distinctly visible. As the bronchial foreign body was removed shortly after aspiration, additional complications beyond atelectasis were prevented.

An increase in dental treatments has led to the development of various artificial dental structures. Although typically checked before endotracheal intubation, they may sometimes be missed. Therefore, rechecking these artificial structures upon ICU admission and extubation may help prevent their aspiration.

In adults, foreign bodies in the airway can be successfully removed using flexible or rigid bronchoscopies. During flexible bronchoscopy, various tools are used to extract foreign bodies from the airway, with forceps being the most common. Forceps are available in multiple configurations to accommodate objects of different sizes and textures, including shark teeth, rat teeth, alligator tips, and rubberized tips. However, our hospital did not have these various types of forceps, and the standard cup forceps used primarily for bronchial biopsy are not as effective for removing airway foreign bodies as the aforementioned forceps. In this case, we attempted to use standard cup forceps for bronchial biopsy before using the cryoprobe; however, the foreign body was too slippery and large to grasp with the cup forceps. We made every effort to remove the dental bridge using flexible bronchoscopy. The balloon, which is typically intended for hemostasis, was used to dislodge the bronchial foreign body tightly lodged in the right main bronchus. The balloon was used to pull the lodged foreign body proximally, and cryoadhesion was used to fix and remove it.

In conclusion, we report a case of bronchial foreign body removal using flexible bronchoscopy with a balloon catheter and cryoadhesions. Selecting appropriate tools based on the characteristics and location of the foreign body is crucial for the successful removal of bronchial foreign bodies. Moreover, rechecking the artificial dental structures upon ICU admission and extubation may help prevent their aspiration.

## Author contributions

**Conceptualization:** Yeonjeong Heo, Jeongwon Heo.

**Investigation:** Yeonjeong Heo.

**Supervision:** Oh Beom Kwon, Da Hye Moon, Seon-Sook Han, Woo Jin Kim, Seung-Joon Lee.

**Writing – original draft:** Yeonjeong Heo.

**Writing – review & editing:** Jeongwon Heo.
